# A novel single‐cell method provides direct evidence of persistent DNA damage in senescent cells and aged mammalian tissues

**DOI:** 10.1111/acel.12573

**Published:** 2017-01-26

**Authors:** Alessandro Galbiati, Christian Beauséjour, Fabrizio d'Adda di Fagagna

**Affiliations:** ^1^ IFOM‐FoundationThe FIRC Institute of Molecular Oncology FoundationVia Adamello 16Milan20139Italy; ^2^ Département de PharmacologieCHU Ste‐JustineMontréalQCH3T 1C5Canada; ^3^ Istituto di Genetica MolecolareConsiglio Nazionale delle RicercheVia Abbiategrasso 20727100PaviaItaly

**Keywords:** aging, cellular senescence, DNA damage, DNA damage response, DNA damage in situ proximity ligation assay, DNA segments with chromatin alterations reinforcing senescence

## Abstract

The DNA damage response (DDR) arrests cell cycle progression until DNA lesions, like DNA double‐strand breaks (DSBs), are repaired. The presence of DSBs in cells is usually detected by indirect techniques that rely on the accumulation of proteins at DSBs, as part of the DDR. Such detection may be biased, as some factors and their modifications may not reflect physical DNA damage. The dependency on DDR markers of DSB detection tools has left questions unanswered. In particular, it is known that senescent cells display persistent DDR foci, that we and others have proposed to be persistent DSBs, resistant to endogenous DNA repair activities. Others have proposed that these peculiar DDR foci might not be sites of damaged DNA per se but instead stable chromatin modifications, termed DNA‐SCARS. Here, we developed a method, named ‘DNA damage *in situ* ligation followed by proximity ligation assay’ (DI‐PLA) for the detection and imaging of DSBs in cells. DI‐PLA is based on the capture of free DNA ends in fixed cells *in situ*, by ligation to biotinylated double‐stranded DNA oligonucleotides, which are next recognized by antibiotin anti‐bodies. Detection is enhanced by PLA with a partner DDR marker at the DSB. We validated DI‐PLA by demonstrating its ability to detect DSBs induced by various genotoxic insults in cultured cells and tissues. Most importantly, by DI‐PLA, we demonstrated that both senescent cells in culture and tissues from aged mammals retain true unrepaired DSBs associated with DDR markers.

## Introduction, Results, and Discussion

DNA double‐strand breaks (DSBs) are among the most cytotoxic forms of DNA damage as failure to repair them leads to genome instability. The DNA damage response (DDR) is a signaling cascade that coordinates DNA repair activities following DNA damage detection and arrests cell cycle progression until lesions have been removed in full (Jackson & Bartek, 2009). Following DSB generation, the apical DDR kinase ATM undergoes activation and phosphorylates the histone H2AX at serine 139; this event, named γH2AX, is necessary for the recruitment of additional DDR proteins to sites of DNA damage, such as the p53 binding protein 1 (53BP1). Therefore, several DDR factors, when activated, are cytologically detectable in the form of nuclear foci assembling at DSB (DDR foci) (Polo & Jackson, 2011). Thus, DNA DSBs can be studied in single cells by immunofluorescence (IF) using antibodies recognizing chromatin modifications (γH2AX) or proteins accumulating in DDR foci (such as 53BP1). However, this may represent a considerable source of bias as, for example, γH2AX may accumulate in the absence of actual DNA damage (Rybak *et al*., 2016; Tu *et al*., 2013). To study DNA breaks in single cells, the only alternatives to IF, at the moment, are terminal deoxynucleotidyl transferase dUTP nick end labeling (TUNEL), which allows DNA ends labeling with fluorescent nucleotides and detection (Shmuel, 1992), and the COMET assay (Olive *et al*., 1991). However, both methods have low sensitivity and are mostly used to detect massive DNA damage, such as that induced by apoptosis.

**Figure 1:**
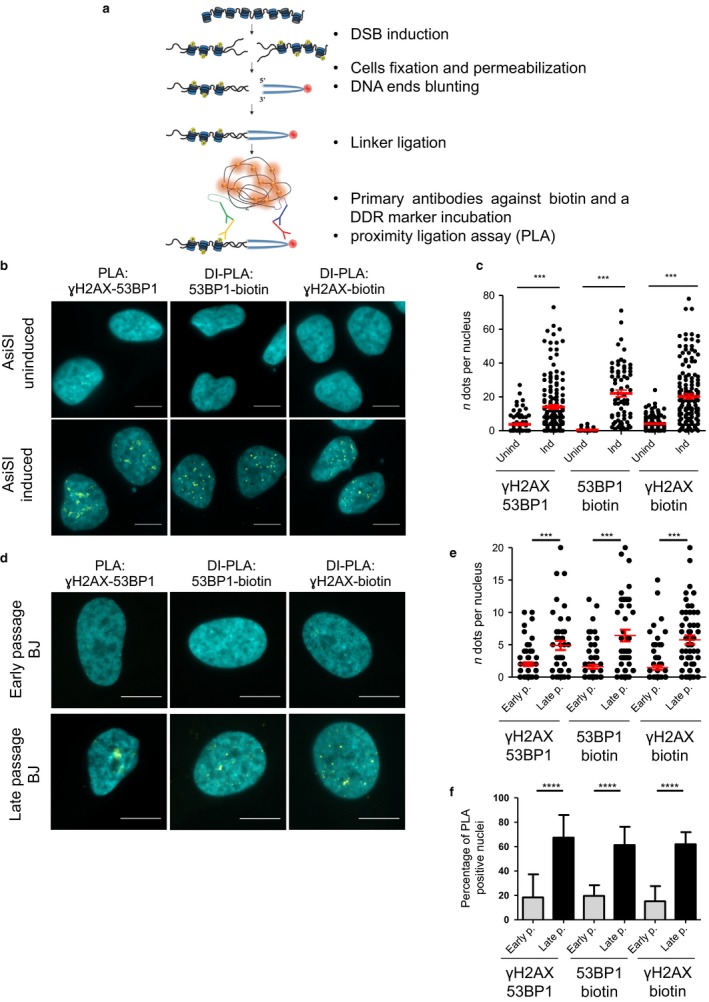
(a) DI‐PLA workflow, see text for details. (b) DSBs generated by AsiSI are detected by DI‐PLA in fixed cells. PLA between ɣH2AX and 53BP1 or DI‐PLA between 53BP1 and biotin or ɣH2AX and biotin, in uninduced (Unind) or induced (Ind) U2OS AsiSI‐ER cells (DNA stained by DAPI). Scale bars: 10 μm. Quantifications are shown in panel (c) (*n* ≥ 3). (d) Replicative senescent cells display unrepaired DSBs as detected by DI‐PLA. PLA between ɣH2AX and 53BP1 or DI‐PLA between 53BP1 and biotin or ɣH2AX and biotin in early passage (Early p.) and senescent (late passage—Late p.) human BJ cells (DNA stained by DAPI). Scale bars: 10 μm. Quantifications are shown in panels (e,f) (*n* ≥ 3).

We therefore developed a novel method, that we named ‘DNA damage *in situ* ligation followed by proximity ligation assay’ (DI‐PLA), that allows the detection and imaging of individual DSBs in a cell. In this protocol, depicted in Fig. 1a, damage‐bearing cells are first fixed by paraformaldehyde (PFA) and permeabilized. This allows DSB ends blunting by *in situ* treatment with T4 DNA polymerase, which has both 3′ overhang resection activity and 5′ overhang fill‐in activity, and subsequent ligation to a biotinylated oligonucleotide (Crosetto *et al*., 2013; Table S1, Supporting information) which permanently tags DNA ends. However, in our hands, the presence of a single biotin molecule at the tagged DSB was not sufficient to generate a signal robustly detectable by IF and standard microscopy (Fig. S1a, Supporting information). To solve this problem, we exploited the power of proximity ligation assay (PLA) which, through rolling circle amplification (RCA), allows high signal amplification (up to 1000‐fold) and sensitivity (Baner *et al*., 1998; Larsson *et al*., 2004). By PLA, when two proteins come in close proximity, ~ 40 nm, and are recognized by primary antibodies, an *in situ* RCA reaction produces a fluorescent signal (dot) detectable at the microscope; it is applicable to both fixed cells and tissues and already used in several studies (Söderberg *et al*., 2006). In DI‐PLA, following the ligation of the biotinylated linker to DNA ends, PLA is performed using one antibody against biotin and a partner antibody against a DDR marker such as γH2AX or 53BP1. Thus, each DI‐PLA signal corresponds to at least one exposed DNA end in close proximity to a DDR marker. Importantly, DDR accumulation in the absence of a DNA end will not generate a DI‐PLA signal.

We first validated DI‐PLA efficiency and specificity in a human cell line expressing an inducible AsiSI restriction enzyme (Iacovoni *et al*., 2010). U2OS AsiSI‐ER cells were induced (or mock‐induced as control), fixed, and treated for IF against individual DDR factors or PLA between them. As shown in Fig. S1b,c (Supporting information) and Fig. 1b,c, both IF and PLA signals between 53BP1 and γH2AX are generated only in induced cells and in similar numbers by both techniques. Then, we performed DI‐PLA between biotin and either 53BP1 or γH2AX (Fig. 1b,c). DI‐PLA nuclear signals were robustly detected in induced cells and not in control cells. Importantly, the number of dots measured by DI‐PLA was very similar between the two sets of antibodies and comparable to that obtained by PLA between 53BP1 and γH2AX in the same conditions (Fig. 1c).

Next, we tested the robustness of DI‐PLA in detecting DSBs generated by different genotoxic treatments, resulting in heterogeneous DSBs. Human BJ fibroblasts were fixed 1 h after exposure to either ionizing radiations (IR), the radiomimetic drug neocarzinostatin (NCS) or mock treatment, and PLA was performed between 53BP1 and γH2AX, while DI‐PLA was performed using antibodies against biotin and either 53BP1 or γH2AX (Fig. S2a–d, Supporting information). By DI‐PLA, we detected signals specifically in damaged cells, with an efficiency comparable to PLA between 53BP1 and γH2AX (Fig. S2a–d, Supporting information) and quantitatively similar to the number of foci measured by IF for 53BP1 or γH2AX (Fig. S2e,f and Fig. S3a,b,d, Supporting information). Moreover, by DI‐PLA, we observed an higher nuclear signal 15 min after IR (Fig. S4a, Supporting information), compared to 1 h after IR, while, increasing IR dose from 2.5 Gy to 5 Gy resulted in a 1.5‐fold increase in DI‐PLA signals, always consistently to similar to what we observed by PLA and IF for DDR markers (Fig. S4a,b, Supporting information). Instead, no signals were detected in the absence of the biotinylated linker, regardless of treatment (Fig. S4c,d, Supporting information). To further validate the dependency of DI‐PLA on DSB, we used an antibody against the histone marker H4 as partner of biotin. While H4 staining resulted in a pan‐nuclear staining unchanged by DNA damaging treatment (Fig. S5a, Supporting information), DI‐PLA between H4 and biotin generated a low background in untreated cells, and a clear increase upon IR, in two different cell lines (BJ and U2OS), and similarly to PLA between H4 and γH2AX (Fig. S5b–d, Supporting information).

Although ionizing radiations are known to induce DSBs with complex end structures, which might inhibit the efficiency of DNA ends blunting by T4 DNA polymerase and reduce DI‐PLA signals, in practice we consistently observed similar results with IF, PLA, and DI‐PLA in all the conditions we tested. Taken together, these results indicate that DI‐PLA reliably detects DSBs generated by different sources, in a dose‐dependent manner, and can thus be used to demonstrate the presence of unrepaired DNA ends in close proximity to activated DDR factors.

When DNA DSBs cannot be repaired in full, unrepaired DNA damage causes persistent DDR activation that enforces a permanent cell cycle arrest termed cellular senescence (d'Adda di Fagagna, 2008). Cellular senescence has been observed *in vivo* in mammals, in association with aging and in the early steps of cancerogenesis (d'Adda di Fagagna, 2008). Senescent cells display persistent DDR foci that are necessary to fuel damage‐induced senescence (Rodier *et al*., 2011). We, and others, have proposed that these are persistent DNA lesions in the form of DSBs that resist cell repair activities (Fumagalli *et al*., 2012; Hewitt *et al*., 2012), based on the fact that such persistent DDR foci are induced by DNA damaging treatments, their morphology is indistinguishable from other DNA damage‐induced foci, and they are preferentially located at the telomeres, where non‐homologous end‐joining DNA repair is inhibited. Others have proposed that such structures might not be sites of damaged DNA per se but instead stable chromatin alterations resulting from damage (without an underlying lesion), which are necessary to reinforce senescence (DNA‐SCARS) (Rodier *et al*., 2011). So far, the lack of an adequate tool to detect the presence or the absence of DNA ends at persistent DDR foci *in situ* has precluded the possibility to conclusively address this question. As DI‐PLA can detect DDR foci only if bearing exposed DNA ends, it is the ideal tool to answer to this long‐standing question.

We compared early (30–32 population doublings) with late‐passage (62–66 population doublings) BJ cells that have undergone replicative senescence, a result of serial passaging that critically shortens telomeres and activates a local DDR (Bodnar *et al*., 1998), as indicated by senescence‐associated β‐galactosidase (β‐gal) activity (Fig. S3f, Supporting information) and reduced 5‐bromodeoxyuridine (BrdU) incorporation after a 6 h pulse (Fig. S3h, Supporting information). Most (~ 85%) of late‐passage BJ cells displayed persistent DDR foci, with a mean of 5 foci per nucleus as determined by IF (Fig. S3a–e, Supporting information). In these same cells, and consistently with what we observed by IF, PLA between 53BP1 and γH2AX generated signals in about 65% of nuclei, with a mean of 5 dots per nucleus; instead, PLA signals could be detected only in a small fraction (20%) of early passage cells, with a mean of 2 dots per nucleus (Fig 1d–f). Having quantitatively established the evidence for persistent DDR activation in replicative senescent cells, we next tested for the presence of DSBs at the persistent DDR foci by DI‐PLA. Strikingly, DI‐PLA between biotin and either 53BP1 or γH2AX generated a 3‐fold increase in average dots per nucleus upon senescence, increasing from 2 in early passage cells to 6 (Fig 1d–f – cytoplasmic signals occasionally observed in senescent cells were not counted). Senescence resulted in DI‐PLA positivity in 60% of cells, in comparison with only 20% in early passage cells.

To strengthen our conclusions, we extended our observations to an additional kind of cellular senescence, the one induced by IR. As previously reported (Fumagalli *et al*., 2012), BJ hTERT cells (obtained by retroviral expression of BJ cells with hTERT) show all features of senescent cells 4 weeks after high‐dose IR, including β‐gal activity (Fig. S3g, Supporting information), reduced BrdU incorporation (Fig. S3i, Supporting information) and persistent DDR foci as visualized by IF for 53BP1 and γH2AX (Fig. S3a–e, Supporting information). In these cells, we performed PLA between 53BP1 and γH2AX and observed that almost 60% of the senescent cells displayed PLA signals with a mean of 5 dots per nucleus, while only 25% of untreated cells were positive for PLA signals, with a mean of 2 dots per nucleus (Fig. S6a–c, Supporting information). We then observed similar results with DI‐PLA between biotin and either γH2AX or 53BP1, with nearly three times more DI‐PLA signals in senescent compared to quiescent cells, consistently with what we had already observed with the other techniques (Fig. S6a–c, Supporting information).

Altogether, the consistent results obtained by IF for the individual DDR markers, PLA between the 53BP1 and γH2AX, and DI‐PLA strongly indicate that the persistent DDR foci detected in senescent cells correspond to genuine DSBs.

**Figure 2:**
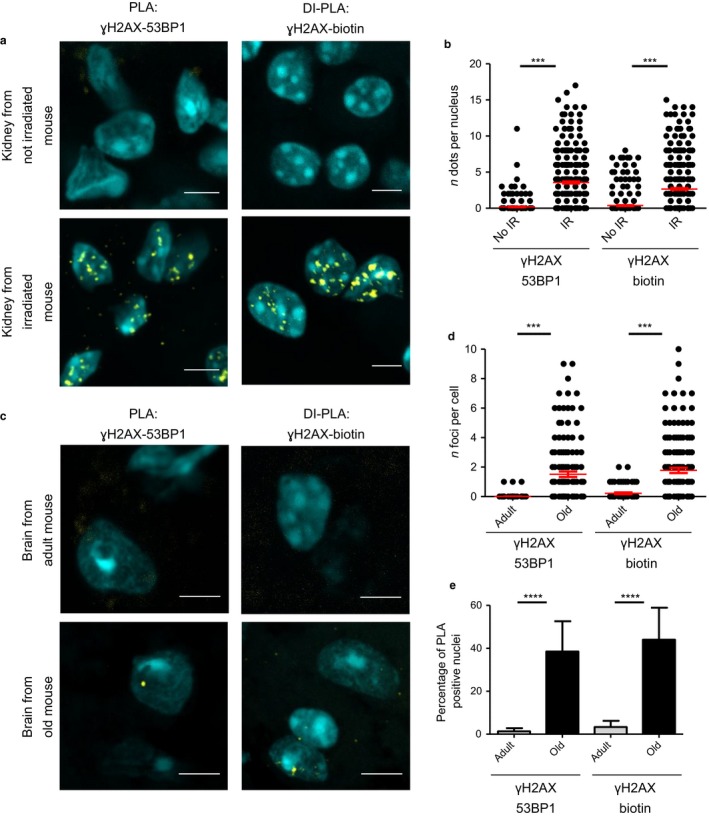
(a) DSBs generated by IR are detected by DI‐PLA in tissue sections derived from mice. PLA between ɣH2AX and 53BP1 or DI‐PLA between ɣH2AX and biotin, in kidney sections from not irradiated (No IR) or irradiated (IR) mice (DNA stained by DAPI). Scale bars: 5 μm. Quantifications are shown in panel (b) (*n* = 3). (c) Aged mammalian tissues display unrepaired DSBs detected by DI‐PLA PLA between ɣH2AX and 53BP1 or DI‐PLA between ɣH2AX and biotin in brain sections from adult (12–14 months) or old (22–24 months) mice (DNA stained by DAPI). Scale bars: 5 μm. Quantifications are shown in panels (d,e) (*n* = 3).

Cellular senescence is considered a major hallmark of organismal aging *in vivo* (López‐Otín *et al*., 2013; Rossiello *et al*., 2014; White *et al*., 2015). Thus, we asked whether we could recapitulate our observations also in tissues from aged animals. To first test the feasibility of DI‐PLA in tissue, we used kidney sections from mice exposed to IR and sacrificed 6 h after treatment, or from untreated mice as a negative control. We detected nuclear signals by DI‐PLA between biotin and γH2AX only in tissue sections from irradiated mice, with an efficiency similar to both PLA between 53BP1 and γH2AX (Fig. 2a,b), and IF for γH2AX (Fig. S7a,b, Supporting information), while, in the absence of the biotinylated linker, DI‐PLA did not generate any detectable signal (Fig. S7c,d, Supporting information).

Having validated DI‐PLA in irradiated tissues, we then asked whether the DDR signals that accumulate in aged tissues correspond to true DSBs. Strikingly, DI‐PLA between biotin and γH2AX generated nearly 10 times more signals in brain sections from old mice (22–24 months) compared to adult mice (12–14 month old) (Fig. 2c–e). The observed 2 DI‐PLA dots per nucleus are very similar to those measured by PLA between 53BP1 and γH2AX under the same conditions (Fig. 2c–e) and previously described in the literature (Sedelnikova *et al*., 2004). We extended these analyses to liver sections of the same aged mice, and, consistently with the aforementioned results, we measured a statistically significant increase with aging in the number of dots generated by DI‐PLA between biotin and γH2AX, although the absolute numbers were overall lower than in the brain (Fig. S7e–g, Supporting information).

Overall, these results indicate that the DDR foci found accumulating in aged tissues correspond to genuine DNA damage.

Recently, several methods (listed in Hu *et al*., 2016) have been developed to detect DSBs in a population of cells. However, they all require high amount of starting material (making them unsuitable for *in vivo* studies) and they are only applicable to study recurrent DSBs (non‐randomly generated). The few alternatives to canonical IF detection to study DNA damage in single cells have poor sensitivity, and thus, they are most commonly used to detect high levels of DNA damage. Here, we propose a novel method, named DI‐PLA, to visualize DNA DSBs at a single‐cell level, which, through the direct tagging of DNA ends, reliably detects only unrepaired DSBs in close physical proximity with an activated DDR protein. By DI‐PLA, we were able to detect DSBs generated by several sources in both cultured cells, and tissues. Most importantly, DI‐PLA allowed us to show for the first time that persistent DDR foci observed accumulating in senescent cells, and aged tissues, correspond to genuine, unrepaired DSBs.

## Funding info

A.G. is supported by Fondazione Italiana per la Ricerca sul Cancro (FIRC, application 16245). F.d'A.d.F.'s laboratory is supported by Associazione Italiana per la Ricerca sul Cancro, AIRC (application 12971), Human Frontier Science Program (contract RGP 0014/2012), Cariplo Foundation (grant 2010.0818), Fondazione Telethon (GGP12059), Association for International Cancer Research (AICR) and an European Research Council advanced grant (322726). C.B. is supported by a grant from the Canadian Institute of Health Research (#MOP‐341566).

## Author contributions

A.G. performed the experiments and generated the data for all the figures C.B. prepared the mice sections used for Fig. 2c–e and Fig. S7e–g (Supporting information). F.d'A.d.F. planned and supervised the project. A.G. and F.d'A.d.F wrote the manuscript. C.B. edited the manuscript.

## Conflict of interest

None declared.

## Supporting information


**Fig. S1** (a) Immunofluorescence for γH2AX and biotin in DNA damaged cells. U2OS AsiSI‐ER cells, DNA damage is induced by the translocation of AsiSI in the nucleus (DNA stained by DAPI). The biotinylated linker has been ligated to exposed DNA ends. Scale bar: 10 μm. (b) Immunofluorescence for γH2AX and 53BP1 in uninduced (Unind) or induced (Ind) U2OS AsiSI‐ER cells (DNA stained by DAPI). Scale bars: 10 μm. Quantification are shown in panels (c) (*n* = 3).
**Fig. S2** (a) PLA between ɣH2AX and 53BP1 or DI‐PLA between 53BP1 and biotin or ɣH2AX and biotin, in not irradiated (No IR) or irradiated (IR) BJ fibroblasts (DNA stained by DAPI). Scale bars: 10 μm. Quantifications are shown in panel (b) (*n* ≥ 3). (c) PLA between ɣH2AX and 53BP1 or DI‐PLA between 53BP1 and biotin, in BJ fibroblasts untreated or treated with NCS for 20 min (DNA stained by DAPI). Scale bars: 10 μm. Quantifications are shown in panel (d) (*n* = 3). (e) Immunofluorescence for 53BP1 in BJ fibroblasts untreated or treated with NCS as in panel (c) (DNA stained by DAPI). Scale bars: 10 μm. Quantifications are shown in panel (f).
**Fig. S3** (a) Immunofluorescence for 53BP1 and ɣH2AX in cells used for PLA and DI‐PLA experiments as in Figs 1d–f, S2a,b and S6 (DNA stained by DAPI). Scale bars: 10 μm. Quantifications are shown in panels (b‐e) (*n* ≥ 3). Late passage BJ fibroblasts are senescent as assessed by β‐gal staining (f) and BrdU incorporation rates (h). IR induces cellular senescence as assessed by β‐gal staining (g) and BrdU incorporation rates (i) in IR‐induced senescent human BJ hTERT fibroblasts SEN (IR). As SEN (IR) cells were contact‐inhibited, cells were replated more sparsely before BrdU incorporation assays. Quiescent (contact‐inhibited) non‐irradiated BJ hTERT fibroblasts (Quie) were used as control.
**Fig. S4** (a) Quantifications for PLA between γH2AX and 53BP1 or DI‐PLA between biotin and γH2AX on U2OS cells untreated (‐IR) or irradiated at the indicated doses and fixed at the indicated time points (*n* = 3). (b) Quantifications for immunofluorescence for γH2AX and 53BP1 on U2OS cells untreated (‐IR) or irradiated at the indicated doses and fixed at the indicated time points (*n* = 2). (c) DI‐PLA between ɣH2AX and biotin in BJ fibroblast not irradiated (No IR) or irradiated (IR) as in Fig S2a,b, in the absence of the biotinylated linker (DNA stained by DAPI). Scale bars: 20 μm. Quantifications are shown in panel (d) (*n* = 2).
**Fig. S5** (a) Immunofluorescence for histone H4 in BJ fibroblasts, untreated (‐IR) or irradiated (IR). (DNA stained by DAPI). Scale bars: 10 μm. (b) Representative images for PLA between γH2AX and 53BP1 or DI‐PLA between gH2AX and biotin, in untreated (‐IR) or irradiated (IR) BJ fibroblasts (DNA stained by DAPI). Scale bars: 10 μm. Quantifications are shown in panel b (*n* = 2). Quantifications for PLA between γH2AX and 53BP1 or DI‐PLA between γH2AX and biotin, in untreated (‐IR) or irradiated (IR) BJ (c) or U2OS (d) cells (*n* = 2).
**Fig. S6** (a) PLA between ɣH2AX and 53BP1 or DI‐PLA between 53BP1 and biotin or ɣH2AX and biotin, in Quiescent (Quie) or IR‐induced senescent (Sen) BJ hTERT fibroblasts (DNA stained by DAPI). Scale bars: 10 μm. Quantifications are shown in panels (b,c) (*n* = 3).
**Fig. S7** (a) Immunofluorescence for ɣH2AX in kidney sections from not irradiated (No IR) or irradiated (IR) mice used for PLA and DI‐PLA experiments as in Fig 2a,b (DNA stained by DAPI). Scale bars: 5 μm. Quantifications are shown in panel (b) (*n* = 3). (c) DI‐PLA between ɣH2AX and biotin, in kidney sections from not irradiated (No IR) or irradiated (IR) mice, in the absence of the biotinylated linker (DNA stained by DAPI). Quantifications are shown in panel (d) (*n* = 2). (e) PLA between ɣH2AX and 53BP1 or DI‐PLA between ɣH2AX and biotin, in liver sections from adult (12–14 months) or old (22–24 months) mice (DNA stained by DAPI). Scale bars: 5 μm. Quantifications are shown in panels (f,g) (*n* = 3).

**Table S1** Sequence of the biotinylated oligonucleotide (ordered from Sigma) used for DI‐PLA experiments.
**Data S1** Experimental procedures.
