# Supplementary figures and images for: A novel single‐cell method provides direct evidence of persistent DNA damage in senescent cells and aged mammalian tissues
Source: Aging Cell. 2017 Jan 26;16(2):422–7. doi: 10.1111/acel.12573 (PMC5334542; doi:10.1111/acel.12573)

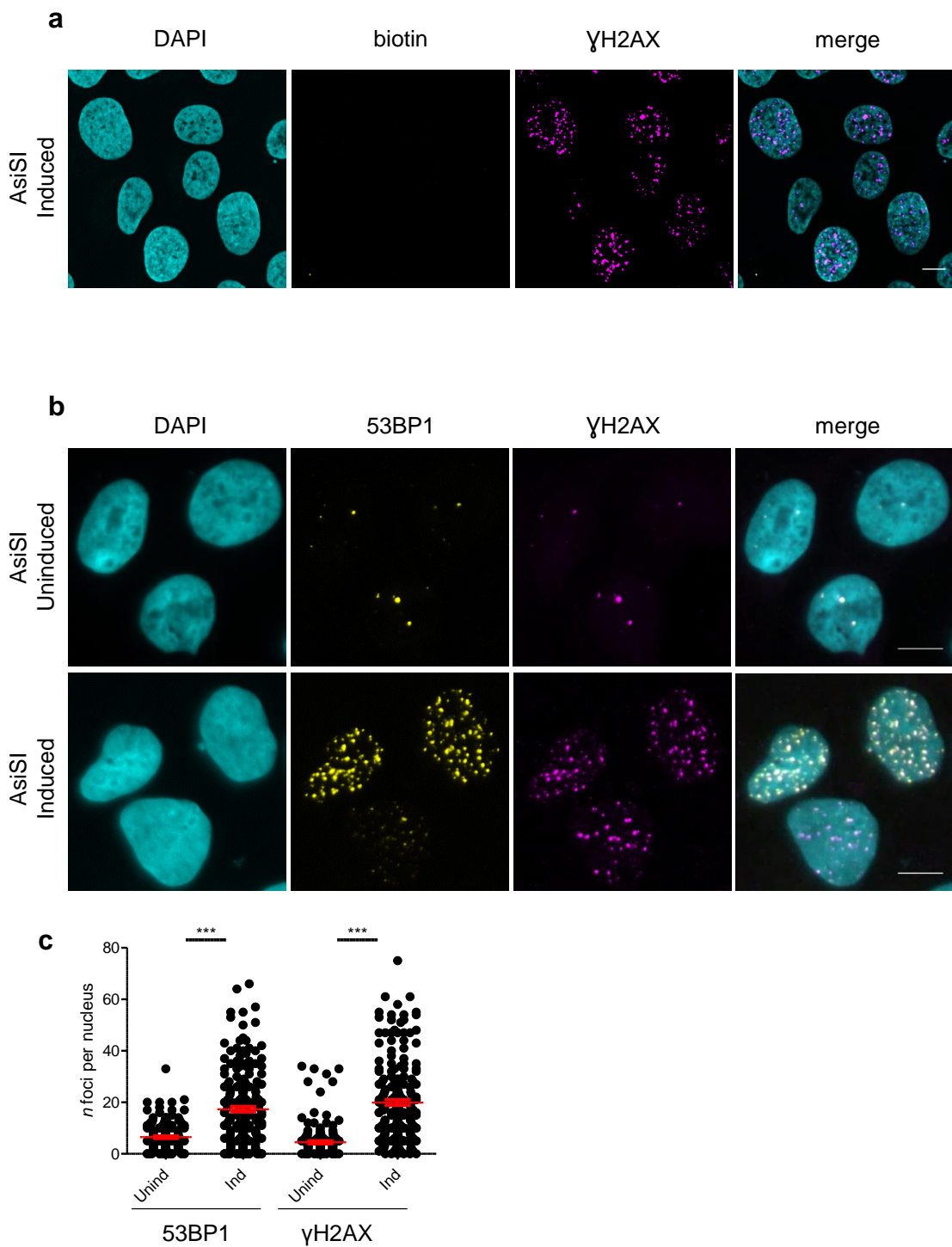

**Figure S1**

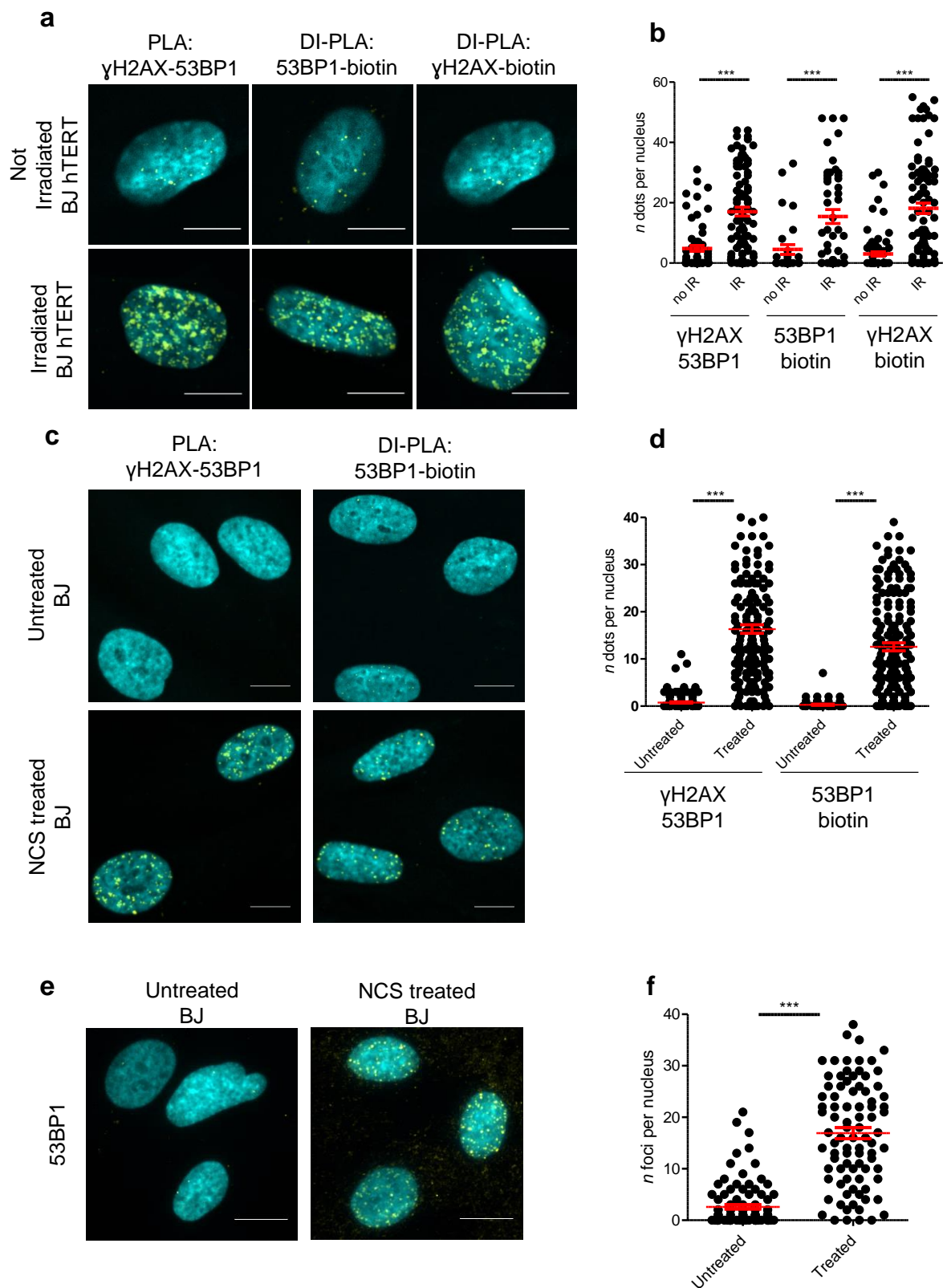

Figure S2

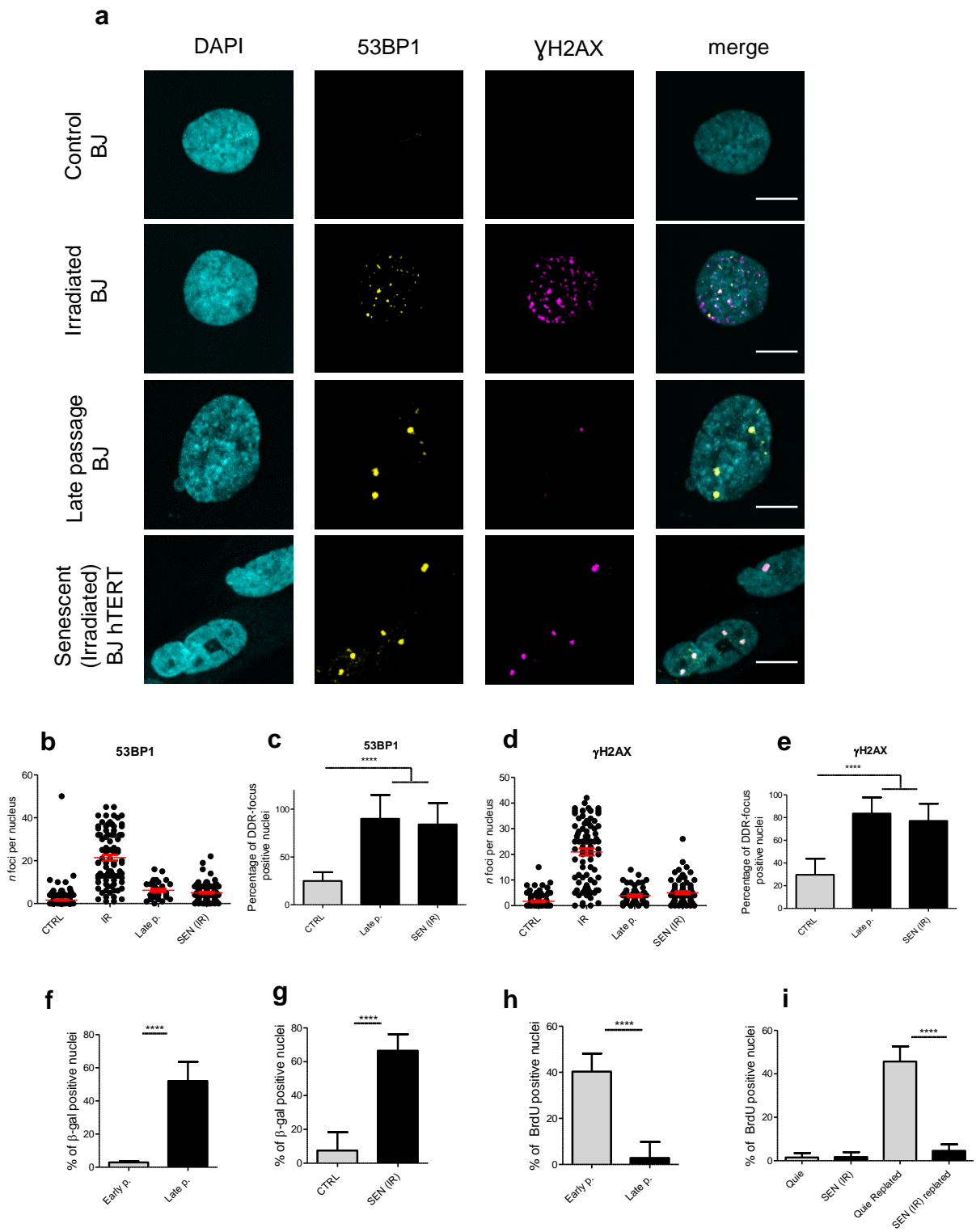

**Figure S3**

**a**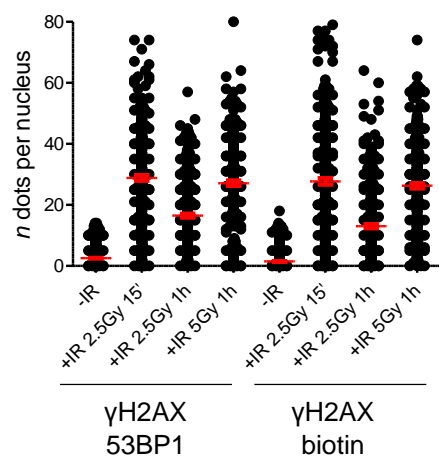**b**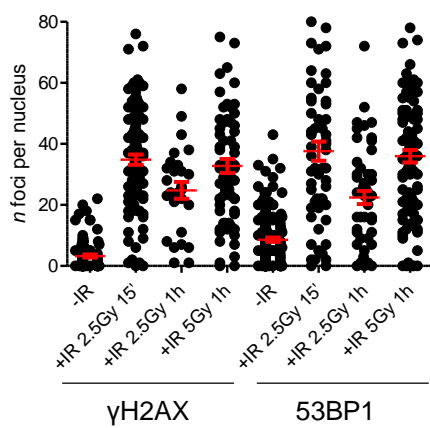**c**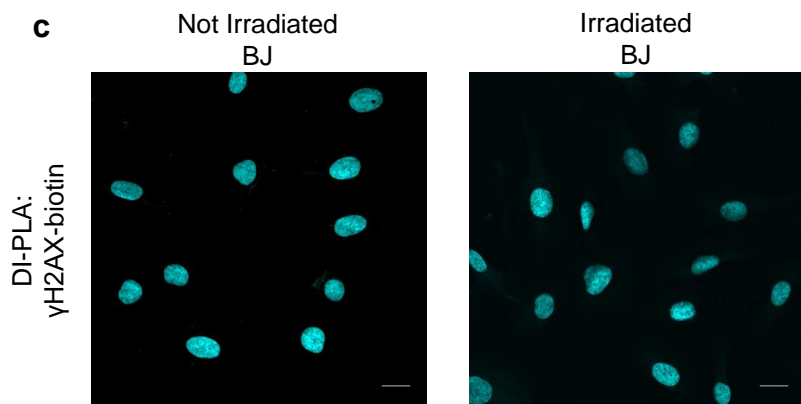**d**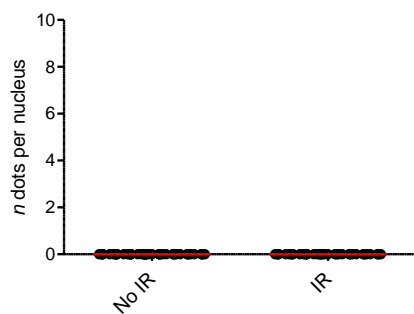**Figure S4**

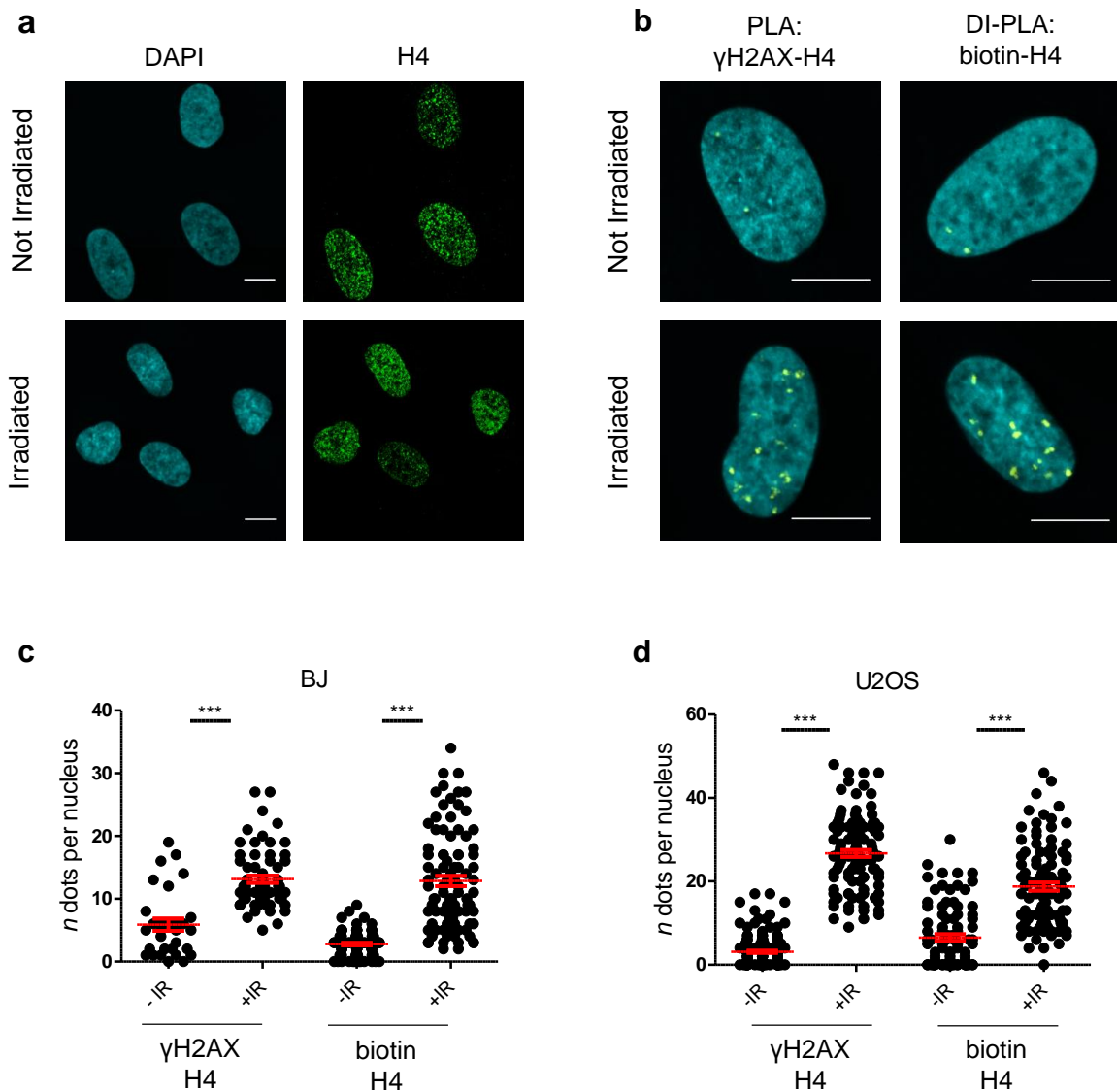

Figure S5

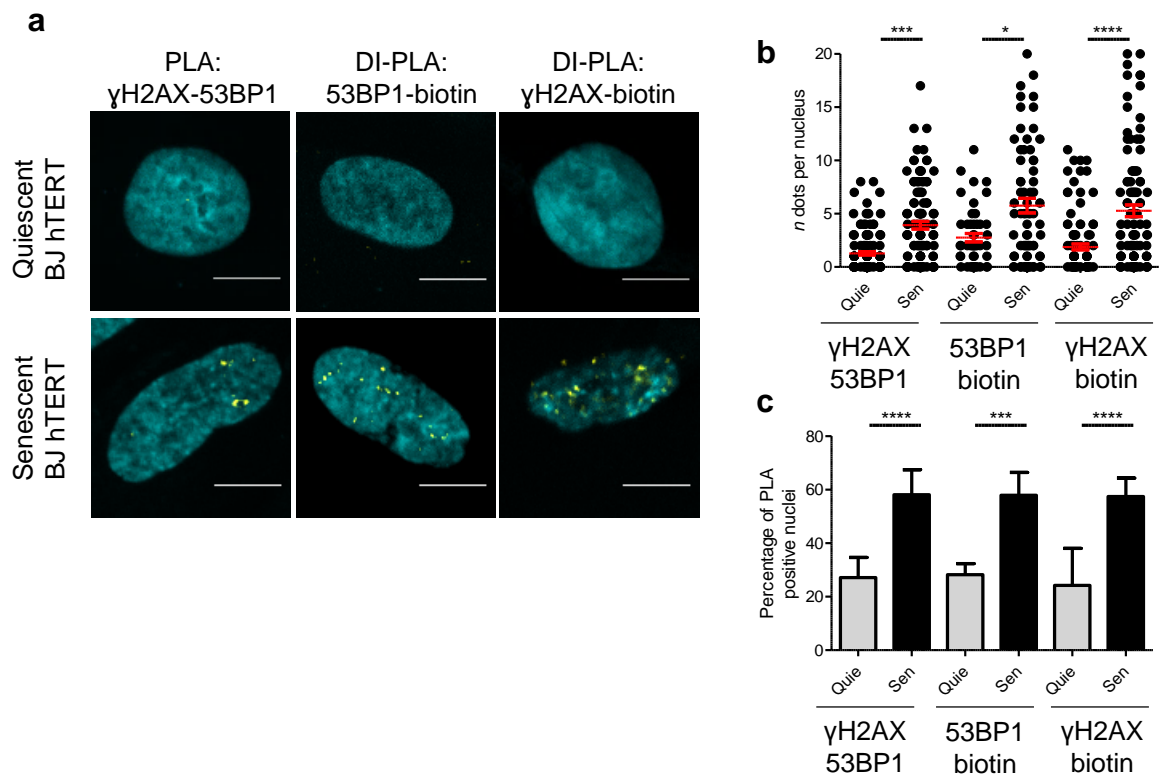

**Figure S6**

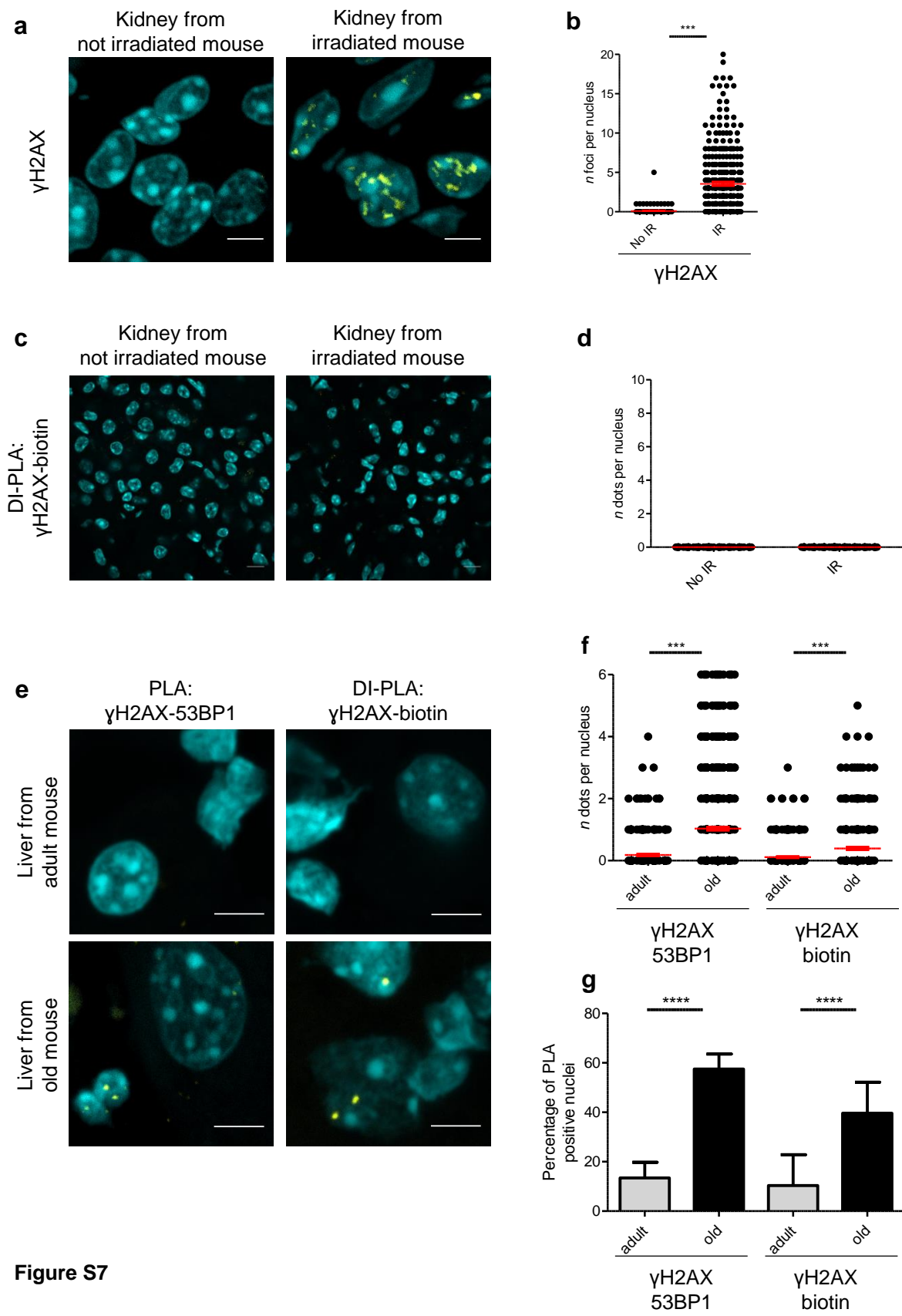

**Figure S7**

Supplement: Supplementary file 1 — Fig. S1 (a) Immunofluorescence for γH2AX and biotin in DNA damaged cells. U2OS AsiSI‐ER cells, DNA damage is induced by the translocation of AsiSI in the nucleus (DNA stained by DAPI). The biotinylated linker has been ligated to exposed DNA ends. Scale bar: 10 μm. (b) Immunofluorescence for γH2AX and 53BP1 in uninduced (Unind) or induced (Ind) U2OS AsiSI‐ER cells (DNA stained by DAPI). Scale bars: 10 μm. Quantification are shown in panels (c) (n = 3). Fig. S2 (a) PLA between ɣH2AX and 53BP1 or DI‐PLA between 53BP1 and biotin or ɣH2AX and biotin, in not irradiated (No IR) or irradiated (IR) BJ fibroblasts (DNA stained by DAPI). Scale bars: 10 μm. Quantifications are shown in panel (b) (n ≥ 3). (c) PLA between ɣH2AX and 53BP1 or DI‐PLA between 53BP1 and biotin, in BJ fibroblasts untreated or treated with NCS for 20 min (DNA stained by DAPI). Scale bars: 10 μm. Quantifications are shown in panel (d) (n = 3). (e) Immunofluorescence for 53BP1 in BJ fibroblasts untreated or treated with NCS as in panel (c) (DNA stained by DAPI). Scale bars: 10 μm. Quantifications are shown in panel (f). Fig. S3 (a) Immunofluorescence for 53BP1 and ɣH2AX in cells used for PLA and DI‐PLA experiments as in Figs 1d–f, S2a,b and S6 (DNA stained by DAPI). Scale bars: 10 μm. Quantifications are shown in panels (b‐e) (n ≥ 3). Late passage BJ fibroblasts are senescent as assessed by β‐gal staining (f) and BrdU incorporation rates (h). IR induces cellular senescence as assessed by β‐gal staining (g) and BrdU incorporation rates (i) in IR‐induced senescent human BJ hTERT fibroblasts SEN (IR). As SEN (IR) cells were contact‐inhibited, cells were replated more sparsely before BrdU incorporation assays. Quiescent (contact‐inhibited) non‐irradiated BJ hTERT fibroblasts (Quie) were used as control. Fig. S4 (a) Quantifications for PLA between γH2AX and 53BP1 or DI‐PLA between biotin and γH2AX on U2OS cells untreated (‐IR) or irradiated at the indicated doses and fixed at the indicated time points (n = 3). [file ACEL-16-422-s001.pdf]
