# Supplementary material for: A novel single‐cell method provides direct evidence of persistent DNA damage in senescent cells and aged mammalian tissues
Source: Aging Cell. 2017 Jan 26;16(2):422–7. doi: 10.1111/acel.12573 (PMC5334542; doi:10.1111/acel.12573)
Supplement: Supplementary file 2 — Table S1 Sequence of the biotinylated oligonucleotide (ordered from Sigma) used for DI‐PLA experiments. Data S1 Experimental procedures. [file ACEL-16-422-s002.doc]

**Supporting information**

**Experimental procedures**

**Cell culture.** U2OS AsiSI-ER cells were grown in Dulbecco’s Modified Eagle Medium (Gibco) containing 10% fetal bovine serum (FBS) phenol red free. Cells were treated with 300 nM 4-Hydroxytamoxifen (4-OHT, Sigma) for 4 h (where indicated).

Early- and late-passage foreskin human fibroblast BJ cells (The American Type Culture Collection), BJ hTERT (obtained by retroviral expression of BJ cells with hTERT), were grown under standard tissue-culture conditions. Confluent cells were fixed and then treated according to the different workflows.

**Animals and treatments.** Mice used to generate Fig. 2a and S7a-d were bred and maintained under pathogen-free condition at the Scripps Research Institute and were handled according to Institutional Animal Care and Use Committee guidelines. Mice received 5Gy TBI and were sacrificed 6h after treatment. Tissues were collected and frozen in dry ice, embedded in OCT tissue TEC (Sakura), mounted on glass slides and stored at -80 °C until use.

Mice used to generate Fig. 2c and S7e (C57BL/6J) were purchased from Charles River Laboratories (Saint-Constant, Quebec); *in vivo* manipulations were approved by the Comité Institutionnel des Bonnes Pratiques Animales en Recherche (CIBPAR) of CHU-Ste-Justine. Immediately after sacrificed, mouse tissues were frozen on dry ice and embedded in OCT compound. Cryosections of 10µm were mounted on glass slides previously treated with 1% gelatin and 0.05% chromo alum, dried at ambient temperature and then stored at −80 °C until use.

**Immunofluorescence.** Cells grown on coverslips were fixed with 4% paraformaldehyde (PFA) in PBS for 10 min and permeabilized with 0.2% Triton for 10 min. Coverslips were blocked in PBG (0.2% cold water fish gelatine, 0.5% BSA in PBS) and incubated with primary antibody. Cells were then washed with blocking buffer and incubated with secondary antibodies. The incubation with secondary antibodies was followed by 3 minutes incubation with DAPI (Sigma). Coverslips were mounted in Mowiol. Images were acquired using a Leica TCS SP2 or AOBS confocal laser microscope by sequential scanning. Optical z-sections at different levels along the optical axis where acquired. In Figure 1b,c and Figure S1 images were acquired with widefield Olympus Biosystems Microscope BX71 and the analySIS or the MetaMorph software (Soft Imaging System GmbH). Comparative immunofluorescence analyses were performed in parallel with identical acquisition parameters. Number of foci per cell were analysed by the imaging software CellProfiler (Carpenter et al. 2006), using the “Speckle counting” pipeline. Same thresholding parameters were used for each sample in the same experiment. Percentages of PLA-positive, DI-PLA-positive or DDR-foci positive cells were scored manually.

**Immunofluorescence on tissue slides.** Tissues sections mounted on glass slides were fixed with 4% PFA in PBS for 20 minutes. All washes were performed with 0.1% Tween in PBS for 5 minutes. Slides were blocked in BSA 2%, Tween 0.1% in PBS and incubated with primary antibodies overnight at +4°C. Tissues were then washed and incubated with secondary antibodies. Nuclei were stained with DAPI (1 μg/ml). Samples were mounted with glycerol solution. Images were acquired with a Leica SP2 or AOBS confocal microscope by sequential scanning. Comparative immunofluorescence analyses were performed in parallel with identical acquisition parameters. Number of foci per cell were analysed by the imaging software CellProfiler (Carpenter et al. 2006), using the “Speckle counting” pipeline. Same thresholding parameters were used for each sample in the same experiment. Percentages of PLA-positive and DI-PLA-positive were scored manually.

**Proximity Ligation Assay (PLA).** Proximity Ligation Assay with selected antibodies was performed according to manufacturer’s instructions (Sigma) with minor modifications. Cells and tissue sections fixation and permeabilization were performed as described for immunofluorescence. For cells experiments PBG (0.2% cold water fish gelatin, 0.5% BSA in PBS) was used as blocking buffer. For tissue experiments 2%BSA 0,1%Tween in PBS was used as blocking buffer and reactions volumes were scaled up in order to completely cover the tissue sections. We used Duolink In Situ PLA Probe Anti-Mouse Plus (Sigma) and Anti-Rabbit Minus (Sigma) in all experiments. We used Duolink In Situ Detection Reagents Orange (Sigma) for all experiments.

**DNA Damage *In situ* ligation Proximity Ligation Assay (DI-PLA).** After fixation and permeabilization as described for immunofluorescence, cells were treated for DI-PLA. Coverslips were washed twice for 5 minutes in Cut Smart buffer (NEB) 1x and once in blunting buffer 1X (NEB). Afterwards, blunting was performed at room temperature for 60 minutes, in a final volume of 50 µL for each coverslip using: 38.5 µL H2O, 5 µL Blunting Buffer 10x (NEB), 5 µL dNTP 1mM (NEB), 0.5 µL BSA, Molecular Biology Grade 20mg/mL (NEB), 1 µL Blunting Enzyme Mix (NEB). Coverslips were then washed twice in CutSmart buffer 1X and twice in T4 Ligase buffer 1X (NEB). Then *in situ* ligation was performed overnight at 16°C in a sealed humid chamber, in 100 µL final volume per coverslip using: 2 µL T4 Ligase (NEB), 2.5 µL 10 uM biotinylated linker (Table S1), 10 µL T4 Ligase Buffer 10X (NEB), 1 µL dATP solution 100mM (NEB), 1 µL BSA, Molecular Biology Grade 20mg/mL (NEB), 83.5 µL H2O. Coverslips were washed twice in PBS and processed as described for PLA.

To perform DI-PLA on tissue slides, reaction volumes were scaled up in order to completely cover the tissue section and we doubled the concentration of biotinylated linker. We found that tissue sections thickness and fixation method might be critical for DNA damage detection by DI-PLA. In particular, we were unable to detect any signals in formalin-fixed, paraffin-embedded (FFPE) tissues. We also occasionally appreciated unequal staining of the tissue sections, probably due to thickness or drying of the reaction mixes.

**Antibodies.** Anti-γH2AX (immunofluorescence, mouse, Millipore, 05-636, 1:1000), Anti-γH2AX (rabbit, Cell Signaling, 9718, 1:1000), Anti-53BP1 (rabbit, Novus Biological, NB100-304, 1:2000). Anti-biotin (mouse, Sigma, B7653, 1:2000), anti-biotin (rabbit, Abcam, AB53494, 1:2000), anti-BrdU (Becton Dickinson, 347580, 1:20), H4 (rabbit, Abcam, AB10158, 1:200).

**Ionizing radiation.** Ionizing radiation was induced by a high-voltage X-ray-generator tube (Faxitron X-Ray Corporation). Cultured cells or mice were irradiated with the indicated doses.

**β-gal assay.** Senescence-associated β-galactosidase assay was carried out as in (Fumagalli et al. 2012).

**BrdU assay.** Cells were labelled with 10 μg ml^−1^ BrdU (Sigma) for 6 h and incorporation was evaluated by immunofluorescence after DNA denaturation.

**Statistical analysis.** Results are shown as mean ± standard error of the mean (s.e.m) or percentages ± 95% confidence interval (c.i.) as indicated. P-value was calculated by Student’s two-tailed t-test or chi-squared test, respectively, using Prism software.

**Reference**

Carpenter AE, Jones TR, Lamprecht MR, Clarke C, Kang IH, et al. 2006. CellProfiler: image analysis software for identifying and quantifying cell phenotypes. Genome Biol. 7(10):R100

**Supplementary figure legends**

**Figure S1 a.** Immunofluorescence for γH2AX and biotin in DNA damaged cells. U2OS AsiSI-ER cells, DNA damage is induced by the translocation of AsiSI in the nucleus (DNA stained by DAPI). The biotinylated linker has been ligated to exposed DNA ends. Scale bar: 10 µm. **b.** Immunofluorescence for γH2AX and 53BP1 in uninduced (Unind) or induced (Ind) U2OS AsiSI-ER cells (DNA stained by DAPI). Scale bars: 10 µm. Quantification are shown in panels **c** (n = 3)**.**

**Figure S2 a.** PLA between ɣH2AX and 53BP1 or DI-PLA between 53BP1 and biotin or ɣH2AX and biotin, in not irradiated (No IR) or irradiated (IR) BJ fibroblasts (DNA stained by DAPI). Scale bars: 10µm. Quantifications are shown in panel **b** (n ≥ 3)**. c.** PLA between ɣH2AX and 53BP1 or DI-PLA between 53BP1 and biotin, in BJ fibroblasts untreated or treated with NCS for 20 minutes (DNA stained by DAPI). Scale bars: 10µm. Quantifications are shown in panel **d** (n = 3)**. e.** Immunofluorescence for 53BP1 in BJ fibroblasts untreated or treated with NCS as in panel **c** (DNA stained by DAPI). Scale bars: 10µm. Quantifications are shown in panel **f.**

**Figure S3 a.** Immunofluorescence for 53BP1 and ɣH2AX in cells used for PLA and DI-PLA experiments as in Figs 1d-f, S2a,b and S6 (DNA stained by DAPI). Scale bars: 10 µm. Quantifications are shown in panels **b-e** (n ≥ 3)**.** Late passage BJ fibroblasts are senescent as assessed by β-gal staining (**f**) and BrdU incorporation rates (**h**). IR induces cellular senescence as assessed by β-gal staining (**g**) and BrdU incorporation rates (**i**) in IR-induced senescent human BJ hTERT fibroblasts SEN (IR). As SEN (IR) cells were contact-inhibited, cells were replated more sparsely before BrdU incorporation assays. Quiescent (contact-inhibited) non-irradiated BJ hTERT fibroblasts (Quie) were used as control.

**Figure S4 a.** Quantifications for PLA between γH2AX and 53BP1 or DI-PLA between biotin and γH2AX on U2OS cells untreated (-IR) or irradiated at the indicated doses and fixed at the indicated time points (n=3). **b.** Quantifications for immunofluorescence for γH2AX and 53BP1 on U2OS cells untreated (-IR) or irradiated at the indicated doses and fixed at the indicated time points (n=2). **c.** DI-PLA between ɣH2AX and biotin in BJ fibroblast not irradiated (No IR) or irradiated (IR) as in Fig S2a,b, in the absence of the biotinylated linker (DNA stained by DAPI). Scale bars: 20µm. Quantifications are shown in panel **d** (n = 2)**.**

**Figure S5** **a.** Immunofluorescence for histone H4 in BJ fibroblasts, untreated (-IR) or irradiated (IR) with ionizing radiation (2Gy). DNA stained by DAPI. Scale bars: 10µm **b.** Representative images for PLA between gH2AX and 53BP1 or DI-PLA between gH2AX and biotin, in untreated (-IR) or irradiated (IR) BJ fibroblasts (DNA stained by DAPI). Scale bars: 10µm. Quantifications are shown in panel b (n = 2). Quantifications for PLA between gH2AX and 53BP1 or DI-PLA between gH2AX and biotin, in untreated (-IR) or irradiated (IR) BJ **(c)** or U2OS **(d)** cells (n=2).

**Figure S6 a.** PLA between ɣH2AX and 53BP1 or DI-PLA between 53BP1 and biotin or ɣH2AX and biotin, in Quiescent (Quie) or IR-induced senescent (Sen) BJ hTERT fibroblasts (DNA stained by DAPI). Scale bars: 10µm. Quantifications are shown in panels **b,c** (n = 3)**.**

**Figure S7 a.** Immunofluorescence for ɣH2AX in kidney sections from not irradiated (No IR) or irradiated (IR) mice used for PLA and DI-PLA experiments as in Fig 2a,b (DNA stained by DAPI). Scale bars: 5µm. Quantifications are shown in panel **b** (n = 3)**. c.** DI-PLA between ɣH2AX and biotin, in kidney sections from not irradiated (No IR) or irradiated (IR) mice, in the absence of the biotinylated linker (DNA stained by DAPI). Quantifications are shown in panel **d** (n = 2)**. e.** PLA between ɣH2AX and 53BP1 or DI-PLA between ɣH2AX and biotin, in liver sections from adult (12-14 months) or old (22-24 months) mice (DNA stained by DAPI). Scale bars: 5µm. Quantifications are shown in panels **f,g** (n = 3)**.**

| **Description** | **Sequence 5'-3'** |
| --- | --- |
| Linker | TACTACCTCGAGAGTTACGCTAGGGATAACAGGGTAATATAGTTT[biodT] TTTCTATATTACCCTGTTATCCCTAGCGTAACTCTCGAGGTAGTA |

**Supplementary Table 1.** Sequence of the biotinylated oligonucleotide (ordered from Sigma) used for DI-PLA experiments.
